# Acupuncture to Treat Primary Dysmenorrhea in Women: A Randomized Controlled Trial

**DOI:** 10.1093/ecam/nep239

**Published:** 2011-03-20

**Authors:** Caroline A. Smith, Caroline A. Crowther, Oswald Petrucco, Justin Beilby, Hannah Dent

**Affiliations:** ^1^Centre for Complementary Medicine Research, University of Western Sydney, New South Wales, Australia; ^2^School of Paediatrics and Reproductive Health, Australian Research Centre for Health for Women and Babies, The University of Adelaide, South Australia, Australia; ^3^School of Paediatrics and Reproductive Health, The University of Adelaide, South Australia, Australia; ^4^Faculty of Health Sciences, The University of Adelaide, South Australia, Australia; ^5^School of Population Health and Clinical Practice, The University of Adelaide, South Australia, Australia

## Abstract

We examined the effectiveness of acupuncture to reduce the severity and intensity of primary dysmenorrhea. A randomized controlled trial compared acupuncture with control acupuncture using a placebo needle. Eligible women were aged 14–25 years with a diagnosis of primary dysmenorrhea. Women received nine sessions of the study treatment over 3 months. The primary outcomes were menstrual pain intensity and duration, overall improvement in dysmenorrhea symptoms and reduced need for additional analgesia, measured at 3, 6 and 12 months from trial entry. A total of 92 women were randomly assigned to the intervention (acupuncture *n* = 46 and control *n* = 46). At 3 months although pain outcomes were lower for women in the acupuncture group compared with the control group, there was no significant difference between groups. Women receiving acupuncture reported a small reduction in mood changes compared with the control group, relative risk (RR) 0.72, 95% confidence interval (CI) 0.53–1.00, *P* = .05. Follow-up at 6 months found a significant reduction in the duration of menstrual pain in the acupuncture group compared with the control group, mean difference –9.6, 95% CI –18.9 to –0.3, *P* = .04, and the need for additional analgesia was significantly lower in the acupuncture group compared with the control group, RR 0.69, 95% CI 0.49–0.96, *P* = .03, but the follow-up at 12 months found lack of treatment effect. To conclude, although acupuncture improved menstrual mood symptoms in women with primary dysmenorrhea during the treatment phase, the trend in the improvement of symptoms during the active phase of treatment, and at 6 and 12 months was non-significant, indicating that a small treatment effect from acupuncture on dysmenorrhea may exist. In the study, acupuncture was acceptable and safe, but further appropriately powered trials are needed before recommendations for clinical practice can be made.

## 1. Introduction

Primary dysmenorrhea refers to painful menstrual periods in the absence of any underlying pathology. Dysmenorrhea is a very common problem among young women, and can occur in up to 50% of menstruating women [1]. There is limited research reporting on the affect of symptoms on women's daily living activities, however studies suggest severe menstrual pain is associated with absence from school or work and restricts other activities of daily life [2, 3].

There are three approaches to the management of primary dysmenorrhea: pharmacological, non-pharmacological and surgical. Studies report simple analgesics are commonly used by young women, with use of analgesics reported by 52% of adolescents, and use of non-steroidal anti-inflammatory drugs (NSAIDs) reported by 42% of adolescents to alleviate their dysmenorrhea [4]. In a Swedish study, 38% of women used analgesics and 22% used oral contraceptives to alleviate their dysmenorrhea [5]. Evidence of efficacy supports use of pharmacological agents such as NSAIDs [6], or the use of oral contraceptives [7] to alleviate menstrual pain, however pain relief may be inadequate for some women, or side effects may not be well tolerated.

Women may use complementary medicines to alleviate their dysmenorrhea, although precise figures describing the frequency of use are not available. Data are limited on patterns of use, but utilization data from the UK indicate acupuncture is used to assist with the management of gynecological or obstetric conditions by 8% of women [8].

Acupuncture has been used to treat dysmenorrhea, and a summary of the evidence from randomized controlled trials (RCTs) is given in two systematic reviews. A Cochrane systematic review published in 2002 included one small trial of acupuncture [9]. A total of 48 women from the USA were allocated to one of four study groups: real acupuncture, placebo acupuncture, standard control and visitation control [10]. No difference in the mean pain scores was found before, during (*P* < .07) or after treatment (*P* < .06) between the study groups. Women in the real acupuncture group showed an improvement in pain relief compared with the placebo acupuncture group (odds ratio (OR) 9.5, 95% confidence interval (CI) 1.7–51.8), the standard control group (OR 16.4, 95% CI 3.2–84.8) and the visitation group (OR 22.0, 95% CI 2.12–117.1). The Cochrane review concluded, “there was insufficient evidence to determine the effectiveness of acupuncture in reducing dysmenorrhea, and that further research was needed” [9]. A recent review included 30 RCTs of acupuncture-related modalities [11]. Total 20 studies compared acupuncture with a control group. Overall trials were of low methodological quality. Nine trials suggested a benefit from acupuncture, however only two trials were of reasonable quality. One of these trials was described earlier in the Cochrane review. A second trial compared acupuncture with ibuprofen and found that acupuncture was more effective for pain relief than ibuprofen (RR 1.20, 95% CI 1.02 to –1.42, *P* < .03) [12]. Nine trials found no differences between groups. Following publication of the recent systematic review, one large trial of acupuncture conducted in Germany randomized 208 women with primary dysmenorrhea to acupuncture or a control (wait list control) [13]. This trial found the intensity of menstrual pain was lower in the acupuncture group compared with the control (RR –2.9, 95% CI –2.9 to –1.6, *P* < .001), and improved quality of life on Short Form 36 (SF36) sub-scales at 3 months was significantly more pronounced in the acupuncture group compared with the control. To summarize, evidence from one recent trial, and two systematic reviews suggests acupuncture may reduce dysmenorrhea. However, the poor methodological quality of most trials suggests a need for further rigorous research.

The aim of our study was to examine the effectiveness of acupuncture in treating primary dysmenorrhea, and thus contribute to the research base of accupunture efficacy. The primary hypotheses of this study were that the use of acupuncture in women with dysmenorrhea compared with control acupuncture would at 3, 6 and 12 months from baseline be effective at (i) reducing symptoms of dysmenorrhea as measured by: reduced severity and duration of pain, reduced need for pain relief and an overall improvement in menstrual symptoms, and (ii) improve quality of life, as measured by improved health status indices, reduce time off work or from school, less restriction on daily life activities and less side effects from treatment. We also examined the acceptability and any adverse effects associated with the use of acupuncture.

## 2. Methods

Women were recruited for the study from the community through their general practitioner, gynecologist or by advertising in the media in South Australia between February 2003 and August 2005. The Ethics Committee of the Women's and Children's Hospital, Adelaide, South Australia, approved the research, and all women gave informed written consent.

Women eligible to join the trial were aged 14–25 years with a diagnosis of primary dysmenorrhea. Our criteria for establishing a diagnosis of primary dysmenorrhea was based on self-reported severe incapacitating pain for at least one day of menses in two menstrual cycles classified by a pre-defined pain score of ≥6/10 on the short form of the McGill questionnaire and a visual analog scale [14], and pain that did not respond well to analgesics. To exclude a diagnosis of secondary dysmenorrhea, women needed to present with evidence of a clinical examination confirming a diagnosis of primary dysmenorrhea, or to attend for an appointment with the study gynecologist. The entry criteria adopted for this trial were based on the inclusion criteria used in the Cochrane systematic review [9]. Women were not eligible if they had secondary dysmenorrhea (defined as identifiable pelvic pathology), or dysmenorrhea associated with an intrauterine device. Women were allowed to continue taking oral contraceptives or pharmacological pain relief if required.

A computer-generated randomization schedule was created using variable block size and stratified by previous use or non-use of acupuncture. The randomization schedule was computer generated by an independent statistician. The allocation of the randomization sequence was concealed centrally by telephone. The telephone randomization service was available 7 days a week at The University of Adelaide, and allocation was made to one of two study groups, acupuncture or control. The randomized treatment allocation was known only to the acupuncturist administering the study intervention.

Three acupuncturists registered with the Australian Acupuncture and Chinese Medicine Association administered the acupuncture to the study groups. Subjects underwent a traditional Chinese medicine (TCM) diagnosis to identify the TCM pattern causing dysmenorrhea. All women received their allocated treatment administered for 30–40 min, weekly for 3 weeks, followed by a week of no treatment during the week of expected menses, for three menstrual cycles. At the end of the treatment phase all women were followed up over a further 3 and 12 months during which no further study treatment was administered. Women were mailed follow-up questionnaires to their home, and the trial co-ordinator telephoned women to encourage response to the follow-up questionnaires.

The design of the acupuncture intervention, and the acupuncture points were based on expert opinion [15], a review of the research undertaken and the experience of the research practitioners. Women in the acupuncture group received acupuncture to treat their underlying TCM diagnosis. The most common diagnoses arising for dysmenorrhea are: stagnation of Qi and blood, stagnation of cold, damp heat attacking the lower jiao, deficiency of Qi and blood and deficiency of kidney and liver. Nine diagnostic patterns with standardized treatment regimens were documented in a treatment protocol. Moxibustion was not administered because of the difficulty of blinding subjects. Seirin 0.2 × 30  mm acupuncture needles were inserted bilaterally to a depth of ≤2 cm, and the needles were retained for 30 min. All subjects received the de qi sensation (which is the needling sensation of soreness, numbness or heaviness) following initial insertion of needles and half way through the treatment session. Reinforcing or reducing stimulation was given according to the TCM diagnoses, and standardized to the individual. Primary acupuncture points include GongSun SP4, Guilai ST29, Zhongji Ren-3, Ciliao BL32, Diji SP8, Sanyinjiao SP6 and additional points used according to the individual diagnoses. A minimum of seven points were used at each treatment. For example,

- Qi and blood stagnation: Taichong LR3, Sanyinjiao SP6, Hegu LI4, Zhongji Ren-3, Ciliao UB32, Xuehai SP10, Qihai Ren-6, Diji SP8, GongSun SP4.
- Qi and blood deficiency: Zusanli ST36, Sanyinjiao SP6, Guanyuan Ren-34, Qihai Ren-6, Geshu BL17, Diji SP8, Pishu UB20, Ciliao UB32.
- Stagnation of cold: Shenshu UB23, Zhongji Ren-3, Qihai Ren-6, Sanyinjiao SP6, Guanyuan Ren-4, Mingmen Du-4, Lie Que LU7, Zhaohai KD6, Zusanli ST36.
- Accumulation of damp heat: Yanglingquan GB34, Quchi LI11, Xingjian LR2, Guilai ST29, Ciliao UB32, Fenglong ST40, Yinlingquan SP9, Shuidao ST28, Sanyinjiao SP6, Sanjiaoshu UB22.
- Kidney and Liver deficiency: Zusanli ST36, Sanyinjiao SP6, Guanyuan Ren-4, Qihai Ren-6, Geshu UB17, Ganshu UB18, Shenshu UB23, Taixi KD3.

The control group received sham acupuncture, with the timing and duration of the control intervention the same as the active acupuncture group. Sham points were not acupuncture points, and the placebo needle was inserted away from classical acupuncture points and meridians on the sacral area, lower back, lower abdomen, foot, lower leg and forearm. These locations ranged 2–4 cm away from an acupuncture point or meridian. Ashi points were not used. Placebo acupuncture needles (0.30 × 30  mm) were used [16]. As the tip of the placebo needle is blunted, skin penetration does not occur. Needles were manually “stimulated" by lifting and thrusting the handle of the needle. Seven bilateral sham points were used.

Primary and secondary endpoints were measured at 3, 6 and 12 months after trial entry. The primary outcome measures specified *a priori*, were menstrual pain intensity and duration measured by the McGill questionnaire [14]. The overall improvement in dysmenorrhea (measured by change in dysmenorrhea symptoms, e.g., nausea, vomiting and mood changes), and the proportion of women requiring additional analgesia for pain relief were gathered by a self-report questionnaire completed by subjects. Subjects completed a self-report questionnaire to collect data on secondary endpoints including restriction with daily living activities, and the SF36 [17]. Data on the acceptability of the treatment intervention, assessment of blinding and the credibility of the intervention were made at the end of the study period [18].

A power analysis showed that a sample size of 92 women would detect a difference in pain reduction of 29%, from 69 to 40% between treatment groups (*P* < .05, 80% power). To detect a change in the SF36 quality-of-life score of 17.7 units from baseline between treatment groups with 80% power at the 5% significance level assuming the standard deviation in SF36 scores was 30, the sample size was 92. We anticipated loss to follow-up would be kept to <15%.

The analyses were carried out by an independent statistician (H.D.) using SAS software, Version 9.2 [19]. The analyses used an “intention to treat" approach. Differences in the primary study outcome measures between the two groups were analyzed using mixed model analysis of variance with subject as a random effect for continuous, normally distributed variables and generalized estimating equations for categorical variables. Adjustments for imbalance between the two treatment-groups were made for body mass index (BMI), smoking and socio-economic status. Differences in secondary outcome measures were analyzed using chi-square or Fisher's exact tests, as appropriate, with log-binomial regression used to adjust for imbalances. A *P*-value of  < .05 was considered to indicate statistical significance (two-sided).

Clinical Trial Registration: Australian New Zealand Clinical Trials Registry, http://www.anzctr.org.au/ ACTRN 12605000766617.

## 3. Results

### 3.1. Trial Recruitment and Subject Flow

**Figure 1:**
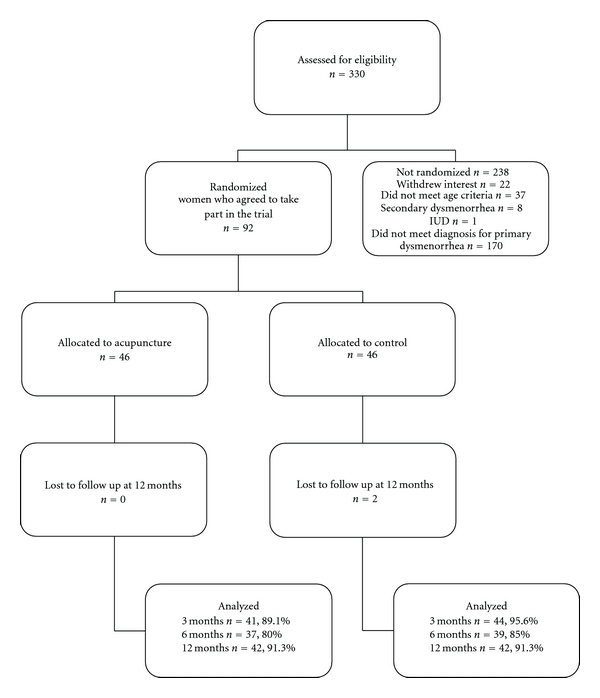
Recruitment and flow of subjects through trial.

Enquiries about the trial were made by 330 women, although just over half (170, 52%) did not meet the criteria for a diagnosis of primary dysmenorrhea based on the pre-specified pain score—one had an IUD, 8 were diagnosed with secondary dysmenorrhea, 37 did not meet the age criteria and 22 withdrew their interest in the study (Figure 1). 

A total of 92 women were randomly assigned to the intervention, with all 46 women in each group included in the analysis. At 3 months data were available from 92% of subjects (acupuncture 89%, control 96%), at 6 months 83% of subjects (acupuncture 80%, control 85%) and at 12 months 91% of subjects (both treatment groups 91%). Two women in the control group were lost to follow-up at 12 months.

### 3.2. Background Characteristics

Most subjects were Caucasians (92%), single (92%) and 57% were students. The median (inter quartile range (IQR)) number of treatments actually received was nine [6–9] for acupuncture and nine [8, 9] for the control group. The randomized groups were comparable for most baseline characteristics at trial entry (Table 1). There was a >5% difference in BMI, smoking and socio-economic indexes for areas (SEIFA) [20] scores and primary outcomes were adjusted for BMI, smoking and SEIFA score in the analyses. 

### 3.3. Primary Outcomes

**Figure 2:**
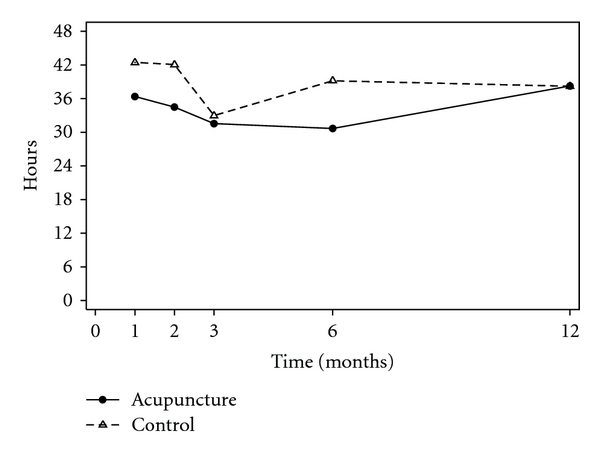
Mean duration of pain (hours).

**Figure 3:**
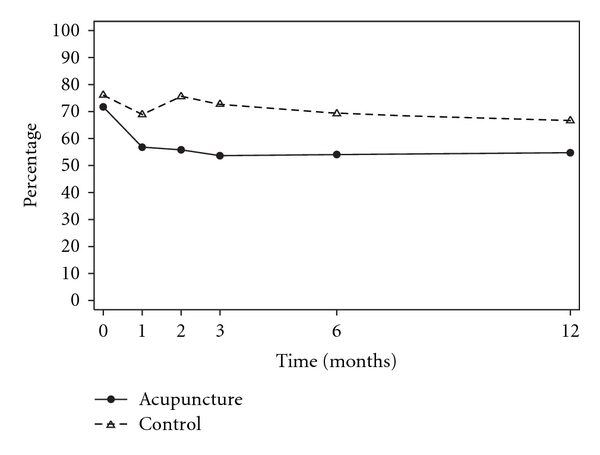
Percentage of women experiencing mood changes.

**Figure 4:**
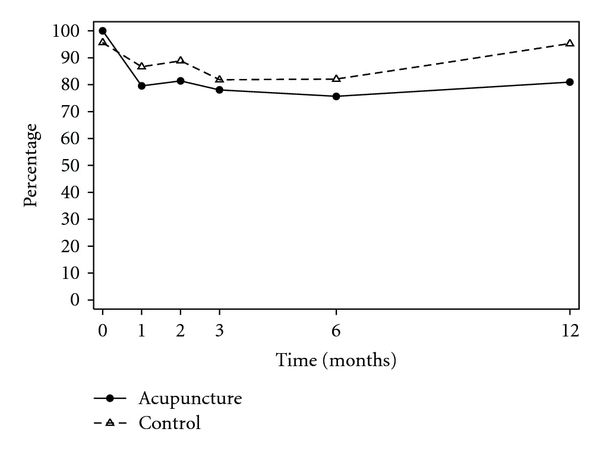
Percentage of women experiencing other menstrual symptoms.

Unadjusted and adjusted analyses are reported for primary outcomes (Table 2). At the end of the 3 months treatment phase although the pain outcomes were marginally lower for women in acupuncture group, there were no significant differences between groups (Figure 2). Women reported fewer mood changes in the acupuncture group (53%) compared with the control group (72%), 0.72, 95% CI 0.53–1.00, *P* = .05, (Figure 3). Six months following trial entry, there was a significant reduction in the duration of menstrual pain in the acupuncture group (30 h) compared with the control group (39 h), *P* = .04, and a reduced need for additional analgesia in the acupuncture (54%) compared with the control group (82%), 0.69, 95% CI 0.49–0.96, *P* = .03. The intensity of pain was lower for women receiving acupuncture, but not significantly different to the control group. At the 12-month follow-up, the earlier improvements reported by women in the acupuncture group were no longer sustained, and there were no differences between groups. The intensity of menstrual pain, duration of pain and the presence of other menstrual symptoms (Figure 4) reduced over time from trial entry for both groups.

### 3.4. Secondary Outcomes

There was no significant improvement in quality of life between the two treatment groups at any time point (Table 3). There were no differences between groups in relation to restriction on daily living activities. 

Subjects were asked to report any side effect or beneficial effects they attributed to “the study intervention" (e.g., dizziness, tiredness, feeling energized, bruising, nausea) at the end of each month. Reporting of side effects or symptoms did not differ between groups. The most frequently reported beneficial effects were feeling relaxed (29%), calm and peaceful (23%). The experience and reporting of any “effect” was highest in the first month of treatment (60% of subjects), and declined over the subsequent 2 months. There were no serious adverse effects reported.

### 3.5. Assessment of Blinding

To assess whether subjects remained blind to their group allocation, women were asked at the end of the third month of the study which group they thought they were allocated to. A total of 41% women receiving acupuncture believed they had received true acupuncture, and 22% of women in the control group thought they were receiving true acupuncture (*P* = .07), 34 and 42%, respectively, were unsure of their group allocation (*P* = .43). Participants' reason for their perceived group allocation were mostly similar between groups, except for 7% of women in the acupuncture group and 29% of women in the control group reporting no improvement (*P* = .01). The trial results indicate that, irrespective of their allocated group, participants liked being in the trial (Table 4). Women liked assisting with the research, the contact with project staff and the attention to their health. A small number of women in the treatment group (*n* = 5, 10%) disliked being in the trial due to the discomfort from needling. There were no differences in responses between groups. 

## 4. Discussion

This study found insufficient evidence that acupuncture administered over a 3-month period provided effective relief from menstrual pain for women with primary dysmenorrhea compared with the control group. Our findings do however suggest that acupuncture improved women's mood symptoms during the treatment phase of the study. Our findings show a non-significant trend in the improvement of menstrual symptoms during the active phase of treatment, and at 6 and 12 months. Evidence of benefit from acupuncture was found in the follow-up period, with a reduction in the duration of pain and less need for pain relief. We used a credible control with subjects unable to identify their group allocation, and our results demonstrate that acupuncture was a safe and acceptable treatment for women. The results suggest that a small treatment effect cannot be excluded, and our study may have been under powered.

Our trial contributes to a body of methodologically rigorous trials of acupuncture to treat dysmenorrhea. Although the evidence from RCTs is encouraging but limited by a small body of high-quality RCTs, evidence of a benefit from acupuncture from uncontrolled study designs grows. A recent observational study found a substantial reduction in pain and the use of NSAIDs by 87% of women, and concluded acupuncture may have a role with the management of dysmenorrhea for women for whom oral contraceptives or NSAIDs are contra-indicated [21]. Other modalities of acupuncture may also be effective with the management of dysmenorrhea. A small pilot study of acupuncture combined with spinal tuina was compared with acupuncture alone [22]. Following 3 months of treatment acupuncture plus tuina appeared more effective than acupuncture alone (93 versus 73%). Zhao reports on a case series of auricular acupuncture, acupoint injection, moxibustion therapy and cupping, all of which suggest a benefit and highlight further areas for research [23].

**Figure 5:**
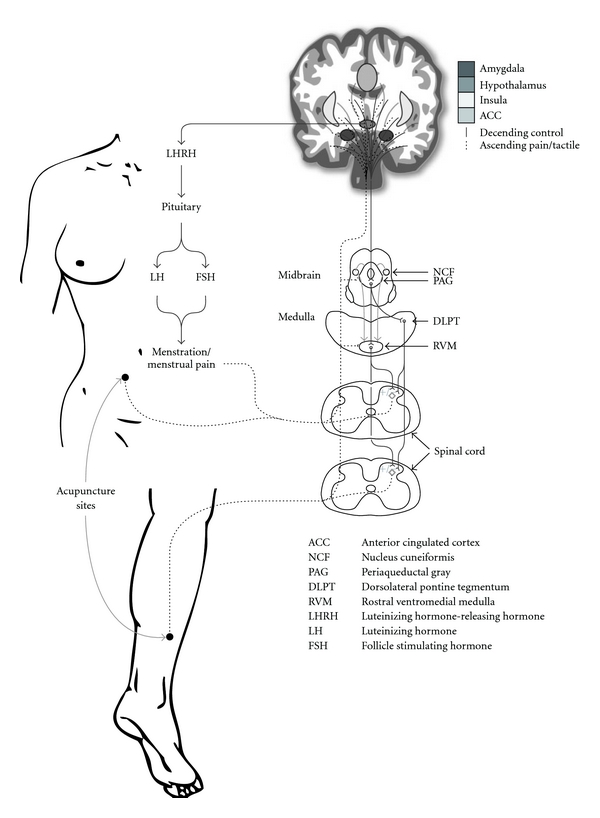
Hypothesized mechanism of acupuncture on dysmenorrhea and related symptoms.

Pain is complex, and a highly individualized experience, that is difficult to assess, examine, manage and treat.  Figure 5 illustrates a potential mechanism of acupuncture for the treatment of dysmenorrhea and symptoms relating to our study findings. Pomeranz proposed that acupuncture stimulation activated A-*δ* and C afferent fibers in muscle [24]. During needle stimulation of acupuncture points such as SP6, SP8 and Ren 4 signals are transmitted to the spinal cord, and via afferent pathways to the midbrain. The perception of pain emerges from the resulting flow and integration of this information among specific brain areas and leads to a change in the perception of pain. The descending pain-modulatory system is a key anatomical network that underlies the ability to change pain intensity [25]. We proposed the brainstem structures involved in the descending modulation alter women's perception of pain resulting from dysmenorrhea. Prostaglandin F2*α* (PGF2*α*) is responsible for dysmenorrhea. Symptoms associated with menstrual pain such as headache, nausea and vomiting, backache and diarrhea can be explained by entry of prostaglandins and prostaglandin metabolites into systemic circulation. The associated improvement in dysmenorrhea-related symptoms we propose are influenced by a subsequent mechanism arising from a release of neurotransmitters in relation to the integration of information from the neural pathways in response to needle stimulation. When these signals reach the hypothalamus and pituitary they trigger a neuro-endocrine response. In an animal model stimulation of acupuncture points CV4, SP6, SP8 have been shown to regulate neuro-endocrine activities including follicle stimulating hormone, luteinizing hormone, estradiol and progesterone [26]. 

Our trial has several strengths. The risk of bias in the trial was minimal, with central randomization, credibility assessment and high compliance with the treatment protocol. We had a low attrition rate at the 6- and 12-month follow-up, and there was no difference in the attrition rate between groups. Overall, the potential for unblinding was low, with women blinded to their group allocation. We consider our acupuncture treatment protocol was representative of what many acupuncture practitioners would administer in practice in relation to selection of acupuncture points, frequency of treatment and the time of needle stimulation, and the treatment protocol was viewed as acceptable by the majority of participants.

The limitations of the trial reflect the lack of power, and whether the results can be representative of women with primary dysmenorrhea. Just >50% of women interested in the study did not meet the inclusion criteria relating to the diagnosis of primary dysmenorrhea based on a self-assessment of menstrual pain. Our results however apply to a smaller group of women with more severe menstrual symptoms. Consideration must also be given to the possibility that the control group was ‘active'. Lund and Lundeberg propose the use of acupuncture controls that involve light touch of the skin such as the placebo needle may stimulate mechanoreceptors that result in the alleviation of the afferent component of pain [27]. This proposition may explain why control interventions are equally effective as acupuncture in alleviating some pain-related conditions [27]. An alternative explanation of the lack of effectiveness of acupuncture in our study at 3 months could attribute the findings to an “inadequate” acupuncture intervention. The treatment protocol used in the effective RCT by Witt [13], delivered the acupuncture intervention over 15 acupuncture sessions (mean number received 10.5 ± 3.1 acupuncture sessions), with practitioners selecting the most appropriate treatment for the individual. Although our trial was shorter in duration, the Witt trial delivered only one additional treatment. The pain outcome measures used in our RCT and the Witt trial used different scales, yet the actual menstrual pain scores at 3 and 6 months appear similar. We suggest the acupuncture intervention in our trial may have been adequate in the main respects including duration, but the lack of effect reflects a lack of power.

It was interesting to observe the optimal effects of acupuncture were experienced 3 months after the study intervention ceased. This observation was also reported in the Witt trial, and may be important for the design of future trials. The finding is difficult to explain from a TCM perspective. The lack of any treatment effect at 12 months following nine treatments was not surprising. In practice, many acupuncture practitioners would administer a tapered schedule of maintenance acupuncture treatments following evidence of a treatment effect. The observation that both groups experienced significant improvements over time lends further support to the overall therapeutic effect of acupuncture comprising in part of non-needling effects benefiting both groups of subjects. The observation of benefit at follow-up warrants further exploration.

Recent research of acupuncture to treat low back pain has found that tailored individualized acupuncture treatments offer no additional benefit over standardized and stimulated acupuncture [28]. The relevance of these findings for acupuncture to treat period pain is unclear, and future research may wish to consider use of both acupuncture individualized, standardized groups in addition to control groups.

Acupuncture was an acceptable and safe treatment, although we found no consistent evidence of benefit from acupuncture. Acupuncture may improve women's mood symptoms but further appropriately powered trials are needed to examine the effect on reducing menstrual pain.

## Figures and Tables

**Table 1:** Characteristics of the women at trial entry by allocated treatment group.

	Acupuncture group, *n* = 46	Control group, *n* = 46
Age in years^a^	19.5 (2.9)	18.9 (3.2)
BMI		
Underweight	4 (8.7)	3 (6.5)
Normal (18.5 to <25)	29 (63.0)	26 (56.5)
Overweight (25 to <30)	6 (13.0)	9 (19.6)
Obese (≥30)	2 (4.3)	4 (8.7)
Missing	5 (10.9)	4 (8.7)
Currently smoking	5 (10.9)	2 (4.3)
Ever alcohol drinking	29 (63.0)	33 (71.7)
Finished high school	10 (21.7)	12 (26.1)
Completed tertiary education	14 (30.4)	13 (28.3)
SEIFA		
Low SEI	14 (30.4)	8 (17.4)
High SEI	15 (32.6)	21 (45.7)
Menstrual pain intensity	5.6 (3.1)	6.1 (2.5)
Other menstrual symptoms	46 (100.0)	44 (95.7)
Need for additional analgesia	43 (93.5)	41 (89.1)
Restricted activities	24 (52.2)	20 (43.5)
SF36		
Physical functioning^b^	95 (70–100.0)	95 (90–100)
Role physical^b^	100 (75–100)	100 (50–100)
Bodily pain	53.8 (20.6)	55.4 (18.7)
General health	68.8 (17.5)	67.7 (21.2)
Vitality	53.7 (20.5)	55.7 (16.3)
Social functioning	75.3 (20.9)	77.2 (19.5)
Role emotional	74.1 (36.9)	75.6 (34.4)
Mental health	63.5 (21.7)	69.4 (14.8)
Overall Physical Component (SF36)	49.0 (8.6)	49.3 (6.8)
Overall Mental component	43.9 (13.1)	45.3 (9.8)
Brazier Health State Utility	0.7 (0.1)	0.81 (0.1)

Values are number (%) of women.

^a^Values are mean (SD), or median (IQR).

^b^SEIFA, a measure of the socio-economic well-being of Australian communities, and identify areas of advantage and disadvantage [19].

**Table 2:** Primary study outcomes by treatment group.

Menstrual outcomes	Acupuncture group, *n* = 46	Control group, *n* = 46	Unadjusted treatment effect (95% CI)	Unadjusted *P*-value	Adjusted treatment effect (95% CI)	Adjusted *P*-value
3 months						
Pain intensity^a^						
Day 1	4.9 (3.2)	4.9 (2.8)	−0.1 (−1.2 to 1.0)	.90	−0.2 (−1.3 to 0.9)	.68
Day 2	3.5 (3.0)	4.0 (2.8)	−0.6 (−1.7 to 0.5)	.28	−0.8 (−1.9 to 0.3)	.17
Day 3	2.0 (2.5)	2.6 (2.7)	−0.6 (−1.7 to 0.5)	.32	−0.7 (−1.8 to 0.4)	.21
Other menstrual symptoms	32 (78.0)	36 (81.8)	0.93 (0.76, 1.15)	.53	0.93 (0.75 to 1.15)	.49
Duration of pain (hours)^a^	31.5 (24.8)	32.9 (19.9)	−1.6 (−10.4 to 7.2)	.72	−2.5 (−11.3 to 6.4)	.59
Need for additional analgesia	26 (63.4)	27 (61.4)	1.01 (0.73 to 1.40)	.96	1.05 (0.76 to 1.44)	.77

6 months						
Pain intensity^a^						
Day 1	4.9 (3.0)	5.0 (2.5)	−0.3 (−1.4 to 0.9)	.67	−0.4 (−1.6 to 0.7)	.47
Day 2	3.8 (3.1)	4.6 (2.7)	−0.9 (−2.1 to 0.2)	.11	−1.1 (−2.3 to 0.0)	.06
Day 3	2.2 (3.0)	2.8 (2.7)	−0.7 (−1.9 to 0.4)	.20	−0.9 (−2.1 to 0.2)	.11
Other menstrual symptoms	28 (75.7)	32 (82.1)	0.93 (0.74 to 1.17)	.53	0.92 (0.74 to 1.15)	.48
Duration of pain (hours)^a^	30.7 (20.9)	39.2 (18.2)	−8.5 (−17.7 to 0.7)	.07	−9.6 (−18.9 to –0.3)	.04
Need for additional analgesia	20 (54.1)	32 (82.1)	0.66 (0.47 to 0.92)	.01	0.69 (0.49 to 0.96)	.03

12 months						
Pain intensity^a^						
Day 1	5.4 (2.7)	4.6 (2.5)	0.8 (−0.3 to 1.9)	.18	0.6 (−0.5 to 1.7)	.29
Day 2	4.8 (2.9)	4.0 (2.4)	0.7 (−0.4 to 1.8)	.20	0.5 (−0.6 to 1.6)	.33
Day 3	2.6 (2.7)	2.8 (2.4)	−0.1 (−1.2 to 1.0)	.81	−0.3 (−1.4 to 0.8)	.60
Other menstrual symptoms	34 (81.0)	40 (95.2)	0.87 (0.75 to 1.02)	.08	0.87 (0.74 to 1.02)	.08
Duration of pain (hours)^a^	38.3 (21.3)	38.2 (21.3)	−1.1 (−9.9 to 7.7)	.81	−2.3 (−11.2 to 6.6)	.62
Need for additional analgesia	34 (81.0)	30 (71.4)	1.13 (0.89 to 1.44)	.31	1.17 (0.92 to 1.47)	.19

Values are number (%) of women.

^a^Values are mean (SD).

**Table 3:** Quality-of-life outcomes by treatment group.

Quality-of-life outcome	Acupuncture group, *n* = 46	Control group, *n* = 46	Unadjusted treatment effect (95% CI)	Unadjusted*P*-value	Adjusted treatment effect (95% CI)	Adjusted *P*-value
3 months (SF36)						
Physical function^a^	95.0 (90.0−100)	95.0 (90.0−100)		.58		.58
Role physical^a^	100 (50−100)	100 (75.0−100)		.81		.96
Bodily pain^b^	64.4 (23.7)	71.9 (21.3)	−6.7 (−15.8 to 2.3)	.14	−6.1 (−15.1 to 2.8)	.18
General health^b^	69.2 (20.5)	66.9 (24.7)	3.2 (−5.8 to 12.1)	.49	5.1 (−3.0 to 13.2)	.22
Vitality^b^	55.8 (21.8)	53.6 (23.1)	1.9 (−6.5 to 10.3)	.66	3.2 (−5.1 to 11.6)	.44
Social function^b^	78.8 (22.3)	79.3 (21.9)	−0.2 (−9.4 to 8.9)	.96	1.1 (−8.0 to 10.3)	.81
Role emotional^b^	75.0 (36.0)	75.0 (33.0)	0.2 (−15.2 to 15.6)	.98	2.2 (−12.7 to 17.1)	.77
Mental health^b^	71.4 (18.2)	67.0 (20.8)	4.5 (−3.3 to 12.2)	.26	6.0 (−1.7 to 13.6)	.13
Overall Physical Component^b^	49.6 (9.1)	52.5 (7.6)	−2.7 (−6.3 to 0.9)	.14	−2.3 (−5.9 to 1.2)	.19
Overall Mental Component^b^	46.1 (10.3)	43.6 (11.8)	2.6 (−2.0 to 7.3)	.26	3.5 (−1.1 to 8.1)	.14
Brazier Health State Utility^b^	0.75 (0.13)	0.75 (0.12)	0.0 (−0.05 to 0.05)	.95	0.0 (−0.04 to 0.05)	.86
Restricted activities	11 (26.8)	14 (31.8)	0.86 (0.44 to 1.68)	.66	0.8 (0.45 to 1.72)	.71

6 months (SF36)						
Physical function^a^	95.0 (85.0−100)	95.0 (90.0−100)		.96		.94
Role physical^a^	87.5 (50−100)	100 (75.0−100)		.41		.62
Bodily pain^b^	69.1 (23.6)	68.3 (21.3)	1.7 (−7.5 to 10.9)	.71	2.5 (−6.7 to 11.6)	.60
General health^b^	69.2 (19.5)	66.3 (25.5)	2.5 (−6.5 to 11.5)	.58	4.5 (−3.7 to 12.7)	.28
Vitality^b^	53.5 (19.8)	56.8 (19.6)	−2.8 (−11.3 to 5.8)	.53	−1.3 (−9.8 to 7.1)	.76
Social function^b^	73.1 (25.4)	80.6 (21.4)	−6.6 (−15.9 to 2.7)	.16	−5.0 (−14.3 to 4.3)	.29
Role emotional^b^	64.6 (38.5)	80.8 (29.1)	−13.8 (−29.4 to 1.8)	.08	−11.3 (−26.5 to 3.8)	.14
Mental health^b^	65.3 (19.2)	72.0 (15.6)	−6.1 (−14.0 to 1.8)	.13	−4.5 (−12.2 to 3.3)	.26
Overall Physical Component^b^	51.5 (9.0)	51.0 (8.6)	1.3 (−2.4 to 4.9)	.50	1.6 (−2.0 to 5.2)	.38
Overall Mental Component^b^	42.4 (11.2)	46.3 (9.8)	−3.9 (−8.6 to 0.8)	.11	−3.0 (−7.6 to 1.7)	.21
Brazier Health State Utility^b^	0.74 (0.10)	0.78 (0.10)	−0.04 (−0.09 to 0.01)	.11	−0.04 (−0.8 to 0.01)	.14
Restricted activities	7 (18.9)	15 (38.5)	0.51 (0.23 to 1.10)	.08	0.50 (0.23 to 1.08)	.08

12 months (SF36)						
Physical function^a^	95.0 (90.0−100)	100 (90.0−100)		.57		.58
Role physical^a^	100 (50.0−100)	100 (50.0−100)		.71		.95
Bodily pain^b^	68.0 (20.7)	73.4 (22.5)	−4.0 (−13.1 to 5.0)	.38	−3.4 (−12.4 to 5.6)	.46
General health^b^	67.6 (19.5)	69.8 (24.0)	−2.8 (−11.7 to 6.1)	.54	−0.9 (−9.0 to 7.3)	.83
Vitality^b^	55.0 (17.7)	56.7 (21.7)	−1.7 (−10.1 to 6.8)	.70	−0.4 (−8.8 to 7.9)	.92
Social function^b^	79.9 (21.8)	81.0 (20.9)	−0.7 (−9.8 to 8.4)	.88	0.7 (−8.4 to 9.8)	.88
Role emotional^b^	69.1 (40.4)	73.0 (35.5)	−5.2 (−20.7 to 10.4)	.51	−2.8 (−17.8 to 12.3)	.72
Mental health^b^	68.5 (15.8)	71.8 (14.2)	−3.3 (−11.1 to 4.5)	.41	−1.8 (−9.4 to 5.9)	.65
Overall Physical Component^b^	51.4 (6.8)	51.7 (10.0)	0.3 (−3.3 to 3.9)	.88	0.6 (−2.9 to 4.2)	.73
Overall Mental Component^b^	44.4 (10.6)	45.5 (8.9)	−1.8 (−6.5 to 2.9)	.44	−1.0 (−5.7 to 3.6)	.67
Brazier Health State Utility^b^	0.76 (0.10)	0.77 (0.11)	−0.01 (0.06 to 0.03)	.62	−0.01 (−0.06 to 0.04)	.72
Restricted activities	19 (45.2)	13 (31.0)	1.46 (0.82 to 2.61)	.20	1.42 (0.83 to 2.44)	.20

Values are number (%) of women.

^a^Values are median (IQR).

^b^Values are mean (SD).

**Table 4:** Secondary study outcomes by allocated treatment group.

	Acupuncture group, *n* = 46	Control group, *n* = 46	Unadjusted treatment effect (95% CI)	Unadjusted *P*-value	Adjusted treatment effect (95% CI)	Adjusted *P*-value
*What I liked*						
I was reassured by the extra attention to my health	22 (47.8)	20 (43.5)	1.10 (0.70–1.72)	.68	1.09 (0.71–1.67)	.70
Few extra demands on my time	4 (8.7)	6 (13.0)	0.67 (0.20–2.21)	.50	—	
My contact with project staff	24 (52.2)	21 (45.7)	1.14 (0.75–1.74)	.53	1.22 (0.81–1.83)	.34
Assisting with research to help others like me	37 (80.4)	33 (71.7)	1.12 (0.89–1.41)	.33	1.12 (0.90–1.39)	.32
There was nothing I liked	0 (0)	1 (2.2)	1.25 (0.36–4.36)	1.0	1.30 (0.36–4.64)	.69
Other	5 (10.9)	4 (8.7)		.73		

*What I disliked*						
I felt more anxious about my health	1 (2.2)	0 (0.0)		1.00	—	
Extra demands on my time	6 (13.0)	6 (13.0)	1.00 (0.35–2.87)	1.00	—	
My contact with project staff	0 (0.0)	0 (0.0)			—	
Being randomized meant I had no say in the decision to have acupuncture	3 (6.5)	3 (6.5)	1.00 (0.21–4.70)	1.00	—	
There was nothing I disliked	32 (69.6)	36 (78.3)	0.89 (0.70–1.13)	.34	0.95 (0.75–1.21)	.67
Other	5 (10.9)	0 (0.0)		.06		

*Would you agree to participate in the research study again?*						
Definitely yes	31 (70.5)	34 (75.6)	0.91 (0.70–1.19)	.49	0.98 (0.75–1.29)	.56
Probably yes	7 (15.9)	9 (20.0)	0.78 (0.32–1.91)	.58	0.74 (0.27–2.03)	.90
I'm not sure	3 (6.8)	1 (2.2)	3.00 (0.32–27.79)	.33		
Probably not	2 (4.5)	1 (2.2)	2.00 (0.19–21.30)	.57		
Definitely not	1 (2.3)	0 (0.0)				

Values are number (%) of women.
